# Initial Presentations Predict Mortality in Pulmonary Tuberculosis Patients - A Prospective Observational Study

**DOI:** 10.1371/journal.pone.0023715

**Published:** 2011-09-13

**Authors:** Jia-Yih Feng, Wei-Juin Su, Yu-Chi Chiu, Shiang-Fen Huang, Yung-Yang Lin, Ruay-Ming Huang, Ching-Hsiung Lin, Jhi-Jhu Hwang, Jen-Jyh Lee, Ming-Chih Yu, Kwok-Woon Yu, Yu-Chin Lee

**Affiliations:** 1 Department of Chest Medicine, Taipei Veterans General Hospital, Taipei, Taiwan, Republic of China; 2 Institute of Clinical Medicine, National Yang-Ming University, Taipei, Taiwan, Republic of China; 3 School of Medicine, National Yang-Ming University, Taipei, Taiwan, Republic of China; 4 Institute of Clinical Medicine and Institute of Brain Science, National Yang-Ming University, Taipei, Taiwan, Republic of China; 5 Laboratory of Neurophysiology and Department of Neurology, Taipei Veterans General Hospital, Taipei, Taiwan, Republic of China; 6 Hua-Lien Hospital, Department of Health, Executive Yuan, Hua-Lien County, Taiwan, Republic of China; 7 Division of Chest Medicine, Department of Internal Medicine, Changhua Christian Hospital, Changhua, Taiwan, Republic of China; 8 Division of Pulmonary and Critical Care, Department of Internal Medicine, Kaohsiung Medical University Chung-Ho Memorial Hospital, Kaohsiung, Taiwan, Republic of China; 9 Department of Internal Medicine, Buddhist Tzu Chi General Hospital, Tzu Chi University, Hualien, Taiwan, Republic of China; 10 Department of Internal Medicine, Wan Fang Hospital, Taipei, Taiwan, Republic of China; 11 Division of Clinical Microbiology, Department of Pathology and Laboratory Medicine, Taipei Veterans General Hospital, Taipei, Taiwan, Republic of China; San Francisco General Hospital, University of California San Francisco, United States of America

## Abstract

**Background:**

Despite effective anti-TB treatments, tuberculosis remains a serious threat to public health and is associated with high mortality. Old age and multiple co-morbidities are known risk factors for death. The association of clinical presentations with mortality in pulmonary tuberculosis patients remains an issue of controversy.

**Methods:**

This prospective observational study enrolled newly diagnosed, culture-proven pulmonary tuberculosis patients from five medical centers and one regional hospital, which were referral hospitals of TB patients. Radiographic findings and clinical symptoms were determined at the time of diagnosis. Patients who died for any reason during the course of anti-TB treatment were defined as mortality cases and death that occurred within 30 days of initiating treatment was defined as early mortality. Clinical factors associated with overall mortality and early mortality were investigated.

**Results:**

A total of 992 patients were enrolled and 195 (19.7%) died. Nearly one-third (62/195, 31.8%) of the deaths occurred before or within 30 days of treatment initiation. Older age (RR = 1.04, 95%CI: 1.03–1.05), malignancy (RR = 2.42, 95%CI: 1.77–3.31), renal insufficiency (RR = 1.77, 95%CI: 1.12–2.80), presence of chronic cough (RR = 0.63, 95%CI: 0.47–0.84), fever (RR = 1.45, 95%CI: 1.09–1.94), and anorexia (RR = 1.49, 95%CI: 1.07–2.06) were independently associated with overall mortality. Kaplan-Meier survival analysis demonstrated significantly higher mortality in patients present with fever (p<0.001), anorexia (p = 0.005), and without chronic cough (p<0.001). Among patients of mortality, those with respiratory symptoms of chronic cough (RR = 0.56, 95%CI: 0.33–0.98) and dyspnea (HR = 0.51, 95%CI: 0.27–0.98) were less likely to experience early mortality. The radiological features were comparable between survivors and non-survivors.

**Conclusions:**

In addition to demographic characteristics, clinical presentations including the presence of fever, anorexia, and the absence of chronic cough, were also independent predictors for on-treatment mortality in pulmonary tuberculosis patients.

## Introduction

Pulmonary tuberculosis (PTB) is an infectious disease with airborne transmission that is associated with high morbidity and mortality worldwide. Despite the advances in anti-tuberculosis (anti-TB) medications and the use of a Direct Observation Therapy/Short Course (DOTS) strategy, the tuberculosis mortality rates remain high in many areas, including Taiwan [1]–[4]. Globally, it has been estimated that tuberculosis causes 1.7 million deaths each year, or about three deaths each minute [1].

Investigating the clinical factors associated with mortality is of paramount importance for the management of PTB patients. The early identification of high risk patients can enable clinicians to provide more aggressive and intensive treatments. Age and underlying co-morbidities have been frequently reported as independent predictors of mortality in previous studies [4]–[6]. By comparison, the extensiveness of radiological presentations and the bacilli loads in sputum have been less frequently mentioned as independent risk factors [7]. Studies that evaluated the impacts of drug susceptibility profiles on mortality also reported controversial results [3], [6]. Most of these predictors of mortality were non-modifiable factors.

Interestingly, the predictive values of the initial presentations of PTB on mortality have rarely been evaluated and have shown inconsistent results [3], [8]–[10]. Although the symptoms/signs of PTB are usually non-specific, typical presentations, such as chronic cough, afternoon fever, and unexplained body weight loss, remain important hints to remind clinical physicians to consider a diagnosis of tuberculosis. In addition, the clinical presentations reflect the interactions between pathogens and host immune responses. The major purpose of the present study was to evaluate the associations of initial presentations for predicting the mortality of PTB patients. The mortality profiles of PTB patients and other clinical predictors of mortality, including bacilli genotyping, were also investigated.

## Materials and Methods

### Ethics

This study was approved by the Institutional Review Board of Taipei Veterans General Hospital; Chest Hospital, DOH, Institutional Review Board; Changhua Christian Hospital Institutional Review Board; Kaohsiung Medical University Chung-Ho Memorial Hospital Institutional Review Board; Taipei Medical University- Wang Fang Medical Center Institutional Review Board; Buddhist Tzu Chi General Hospital and Tzu Chi University, Hualien, Research Ethics Committee; and Chest Hospital, Department of Health, Executive Yuan, Research Ethics Committee. Written informed consent was obtained from each patient or their authorized representative(s) before enrollment.

### Patients and setting

This prospective observational study was conducted at six hospitals in Taiwan: five referral medical centers and 1 regional hospital specializing in pulmonary diseases. Newly diagnosed, culture-proven tuberculosis patients from January 2007 to June 2009 were eligible for enrollment. Patients without pulmonary involvement and those who were younger than 18 years of age were excluded. Demographic profiles (age, gender, co-morbidities) and clinical characteristics (history of previous anti-TB treatments, and smoking habits) were obtained from the patients by enrollment interview. The presenting symptoms/signs were determined according to the subjective statements of the patients and/or their caregivers in critical ill patients. In patients with altered mental status, the presence of dyspnea was determined when the respiratory rate excessed 25/minute. Drug susceptibility profiles were collected from medical records.

Initial presentations were divided into pulmonary symptoms/signs (chronic cough lasting longer than 3 weeks, hemoptysis, and dyspnea) and constitutional symptoms/signs (body weight loss, fever, malaise, and anorexia). Chest radiographs were reviewed by the doctor in-charge at each hospital. Patients were categorized as having cavitation, lobar/segmental consolidation, or bilateral disease if these were present on chest radiographs at the time of diagnosis.

### Treatment of pulmonary tuberculosis and follow-up

All of the patients were treated with standard anti-TB treatments that included isoniazid, rifampicin, ethambutol, and pyrazinamide in the initial phase. The regimen was modified when drug susceptibility results were available or when significant adverse effects occurred. All of the patients were followed until death or upon completion of anti-TB treatment. The treatment outcome was determined according to the records in the Centers for Disease Control (CDC) registration database, Taiwan. Patients who died for any reason before or during the course of anti-TB treatment were defined as mortality cases and mortality that occurred within 30 days of initiating PTB treatment was defined as early mortality [11] Defaulted cases and patients who were transferred out were excluded from analysis.

### Mycobacteriology and genotyping

Sputum AFB smears and cultures were performed in each hospital using standard methods. Sputum smears were examined through Ziehl-Neelsen staining. The isolation of MTB in sputum cultures was performed in liquid medium (BACTEC) and/or Lowenstein-Jensen solid medium. Drug susceptibility testing was performed using the proportion method with Middlebrook 7H11 media. Genomic DNA was extracted from cultures as described previously [12]. All clinical isolates were genotyped using a commercial spoligotyping kit (Isogen Bioscience B.V., Maarssen, Netherlands). The “Beijing strain” was defined as a deletion from spacer 1 to spacer 34 in the direct repeat region and the presence of (at least 3) spacers in 35–43.

### Statistical analysis

The cumulative incidence of mortality was estimated by Kaplan-Meier analysis. The statistical significances and risk ratios of overall and early (deaths within 30 days) mortality by clinical characteristics were analyzed using unadjusted and adjusted Poisson regression analysis with a stepwise selection procedure. The proportional-hazards assumption was tested on the basis of Schoenfeld residuals after a fitted Cox regression model. For time varying covariates, i.e. the hazard ratios for dying varied with follow-up time, averaged hazard ratios were reported. A *p* value of < 0.1 in unadjusted model was required for a variable to be entered into the adjusted model. Variables were included in the final model for p<0.05 and were excluded if p<0.1. Survival time was estimated by the Kaplan-Meier method and a log-rank test was used to evaluate factors associated with death. The patients of defaults and transferred out were censored in the Kaplan-Meier survival analysis. A *p* value of < 0.05 (two-tailed) was considered statistically significant for all tests. Statistical analysis used SPSS version 14.0 (SPSS Inc., Chicago, IL, USA) and STATA 11.0 (Stata corporation, College Station, Texas, USA).

## Results

### PTB Patient Mortality

**Figure 1:**
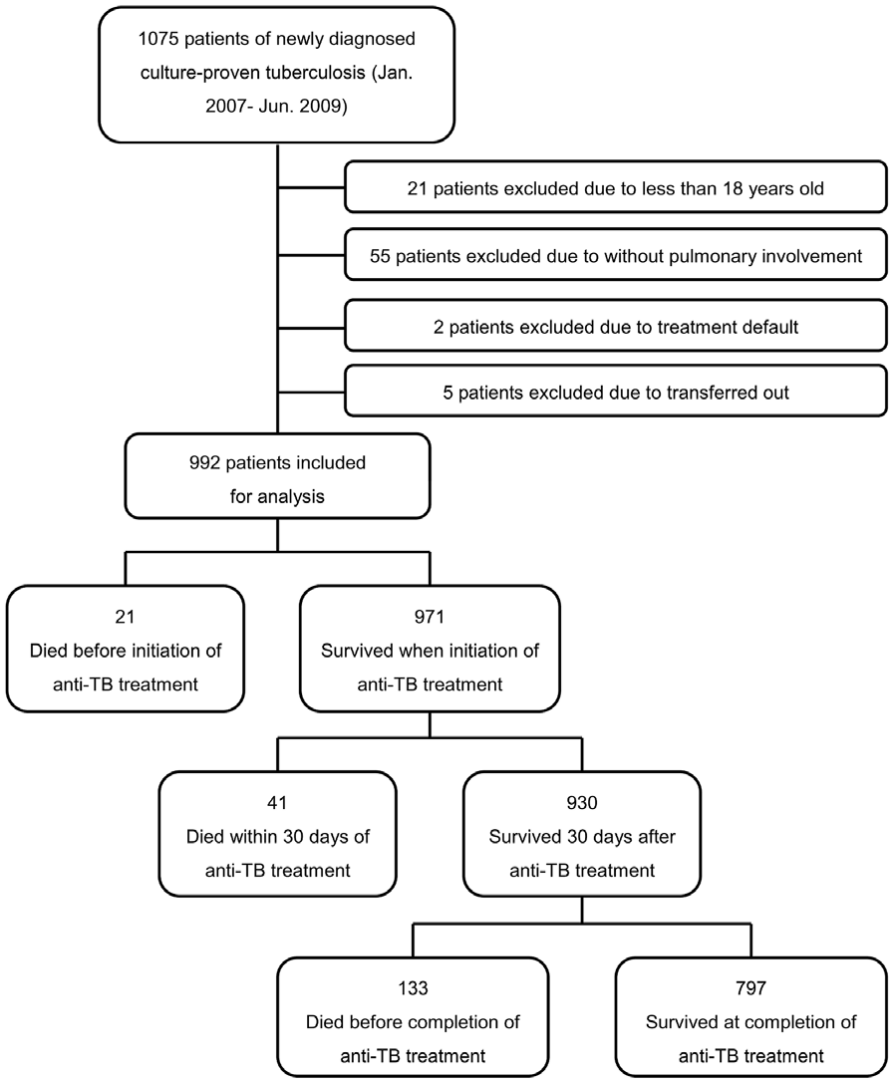
Study profile demonstrating the number of cases and reasons for exclusion.

The study profile showing the number of cases and reasons for exclusion is shown in Figure 1. Ultimately, 992 patients with newly diagnosed, culture-proved pulmonary tuberculosis were included for analysis.

**Figure 2:**
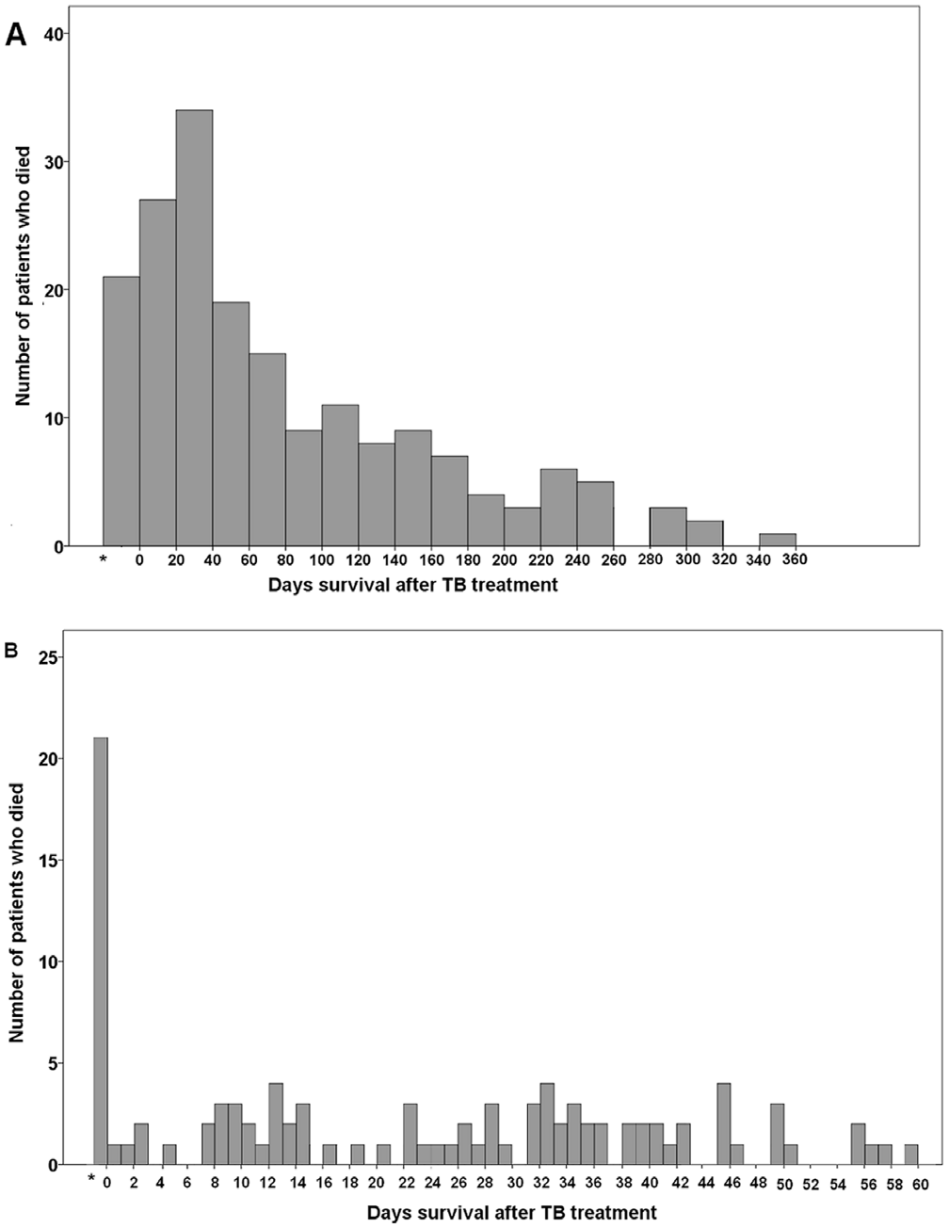
Survival time of pulmonary tuberculosis patients after initiation of anti-TB treatment. (A) within one year. (B) within 60 days. * Number of cases died before initiation of anti-TB treatment.

Overall, 992 patients were followed up for 646.8 person-year. Of these patients, 62 (6.3%) died within 30 days, 185 (18.6%) died within one year, and 195 (19.7%) died before completing treatment. The 30-day and one-year cumulative mortality was 6.25% (95% CI:4.91%–7.94%) and 22.55% (95% CI: 18.95%–26.71%), respectively. The survival times for PTB patients in the first 60 days and within one year are shown in Figure 2. The first 40 days had the largest numbers of mortality cases, and these numbers gradually declined thereafter.

All the 21 pre-treatment mortality cases were diagnosed by positive sputum culture results and enrolled before mortality. However, the anti-TB treatment was not started immediately after diagnosis and the patients died before initiation of treatment. The delay in anti-TB treatment was mostly due to concomitant hepatic and renal dysfunction, inability to take oral medications, or by clinical judgment of the in charge physicians. The intervals from diagnosis to mortality ranged from 13 days to 1 day in these patients.

### Clinical Presentations of Survivors and Non-survivors

The demographic characteristics and clinical presentations of the study population are shown in Table 1. Their mean age was 64.6 (±19.2) years and they were predominantly males (77.6%). There were 13 (1.3%) HIV-positive patients. Compared with survivors, non-surviving patients were more likely to be male (85.6% vs. 75.7%, p = 0.003), older in age (76.2±12.6 vs. 61.5±19.5, p<0.001), have some malignancy (31.3% vs. 8.9%, p<0.001), have renal insufficiency (11.8% vs. 3.3%, p<0.001), and were post-gastrectomy (7.2% vs. 2.1%, p<0.001). The proportions of HIV-positive patients were comparable between survivors and non-survivors, and there were no differences in first-line anti-TB drug resistance rates. The proportions of Beijing strain infections were similar between the two groups.

**Table 1:** Demographic characteristics and clinical presentations of pulmonary tuberculosis patients with or without on-treatment mortality. a

	All patients, n = 992	Survival status		P value
		Survivors, n = 797	Non-survivors, n = 195	
Mean age (SD)	64.6 (19.2)	61.5 (19.5)	76.2 (12.6)	<0.001
Male gender	770 (77.6%)	603 (75.7%)	167 (85.6%)	0.003
Previous TB history	94 (9.5%)	76 (9.5%)	18 (9.2%)	0.90
Smoking habit	297 (29.9%)	248 (31.1%)	49 (25.1%)	0.10
Initial sputum smear positive	484 (48.8%)	393 (49.3%)	91 (46.7%)	0.51
Concomitant extrapulmonary TB	38 (3.8%)	30 (3.8%)	8 (4.1%)	0.83
Comorbid diseases				
Diabetes	223 (22.5%)	175 (22.0%)	48 (24.6%)	0.43
COPD	75 (7.6%)	55 (6.9%)	20 (10.3%)	0.11
Malignancy	132 (13.3%)	71 (8.9%)	61 (31.3%)	<0.001
Renal insufficiency	49 (4.9%)	26 (3.3%)	23 (11.8%)	<0.001
Liver cirrhosis	34 (3.4%)	25 (3.1%)	9 (4.6%)	0.31
HIV positive b	13 (1.3%)	12 (1.5%)	1 (0.5%)	0.28
Post gastrectomy	31 (3.1%)	17 (2.1%)	14 (7.2%)	<0.001
Drug susceptibility test				
Isoniazid resistance	121 (12.2%)	98 (12.3%)	23 (11.8%)	0.85
Rifampicin resistance	65 (6.6%)	54 (6.8%)	11 (5.6%)	0.57
Ethambutol resistance	73 (7.4%)	59 (7.4%)	14 (7.2%)	0.92
Streptomycin resistance	111 (11.2%)	93 (11.7%)	18 (9.2%)	0.33
MDR	59 (5.9%)	49 (6.1%)	10 (5.1%)	0.59
Beijing strain infection	545 (54.9%)	433 (54.3%)	112 (57.4%)	0.43
Radiographic presentations				
Cavity formation	186 (18.8%)	164 (20.6%)	22 (11.3%)	0.003
Lobar/segmental consolidation	807 (81.4%)	637 (79.9%)	170 (87.2%)	0.020
Bilateral involvement	413 (41.6%)	316 (39.6%)	97 (49.7%)	0.010
Respiratory symptoms				
Chronic cough	581 (58.6%)	498 (62.5%)	83 (42.6%)	<0.001
Hemoptysis	167 (16.8%)	132 (16.6%)	35 (17.9%)	0.64
Dyspnea	238 (24.0%)	179 (22.5%)	59 (30.3%)	0.022
Constitutional symptoms				
Body weight loss	213 (21.5%)	180 (22.6%)	33 (16.9%)	0.08
Fever	301 (30.3%)	218 (27.4%)	83 (42.6%)	<0.001
Malaise	147 (14.8%)	106 (13.3%)	41 (21.0%)	0.006
Anorexia	179 (18.0%)	129 (16.2%)	50 (25.6%)	0.002

a The data are presented as n (%) unless otherwise stated.

b HIV testing was not routinely done in each patient. The estimated HIV testing rate ranged around 15–20%.

TB, tuberculosis; COPD, chronic obstructive pulmonary disease; HIV, human immunodeficiency virus; MDR, multi-drug resistance; RR, risk ratio; CI, confidence interval.

Regarding radiological features, the non-survivors were less likely to have cavity formations (11.3% vs. 20.6%, p = 0.003), and were more likely to have lobar/segmental consolidations (87.2% vs. 79.9%, p = 0.02) and bilateral involvements (49.7% vs. 39.6%, p = 0.01). Regarding clinical symptoms/signs, the non-survivors were less likely to present with chronic cough of > 3 weeks (42.6% vs. 62.5%, p<0.001), and were more likely to present with dyspnea (30.3% vs. 22.5%, p = 0.022), fever (42.6% vs. 27.4%, p<0.001), malaise (21% vs. 13.3%, p = 0.006), and anorexia (25.6% vs. 16.2%, p = 0.002). Multivariate Poisson regression analysis identified age, malignancy, renal insufficiency, chronic cough, fever, and anorexia as independent predictors associated with overall mortality.

Independent risk factors associated with overall mortality from a multivariate Cox proportional hazards model are summarized in Table 2. Older Age (HR = 1.05, 95% CI: 1.04–1.06), malignancy (HR = 2.85, 95% CI: 2.08–3.91), renal insufficiency (HR = 2.27, 95% CI: 1.43–3.59), absence of chronic cough > 3 weeks (HR = 0.54, 95% CI: 0.40–0.72), presence of fever (HR = 1.72, 95% CI: 1.29–2.30), and anorexia (HR = 1.49, 95% CI: 1.07–2.07) were independent predictors of mortality.

**Table 2:** Cox proportional hazards models for overall mortality prediction by demographic characteristics and clinical presentations.

	Unadjusted HR (95% CI) a		Adjusted HR (95% CI) b	
	HR (95% CI)	*p* value	HR (95% CI)	*p* value
Age	1.06 (1.05–1.07)	<0.001	1.05 (1.04–1.06)	<0.001
Male gender	1.78 (1.19–2.66)	0.005		
Malignancy^c^	3.59 (2.65–4.87)	<0.001	2.85 (2.08–3.91)	<0.001
Renal insufficiency	3.33 (2.16–5.16)	<0.001	2.27 (1.43–3.59)	<0.001
Post gastrectomy	3.03 (1.76–5.23)	<0.001		
Cavitary lesion	0.47 (0.30–0.73)	0.001		
Lobar/segmental consolidation	1.77 (1.16–2.71)	0.008		
Bilateral involvement	1.44 (1.08–1.90)	0.012		
Chronic cough	0.46 (0.34–0.61)	<0.001	0.54 (0.40–0.72)	<0.001
Dyspnea	1.40 (1.03–1.90)	0.031		
Fever	1.82 (1.37–2.42)	<0.001	1.72 (1.29–2.30)	<0.001
Malaise	1.54 (1.09–2.18)	0.014		
Anorexia	1.60 (1.16–2.21)	0.004	1.49 (1.07–2.07)	0.018

a Unadjusted hazard ratios and 95% confidence intervals for variables that might predict overall mortality (*p*<0.1).

b Results of stepwise selection for a fitted cox regression model (Goodness-of-fit, Chi square =  214.226, *p*<0.001). Variables were included in the final model for a p<0.05 and were excluded if p<0.1.

c The hazard of dying for patients with malignancy or dyspnea attenuated after 6 months and averaged hazard ratios were reported.

HR, hazard ratio; CI, confidence interval.

**Figure 3:**
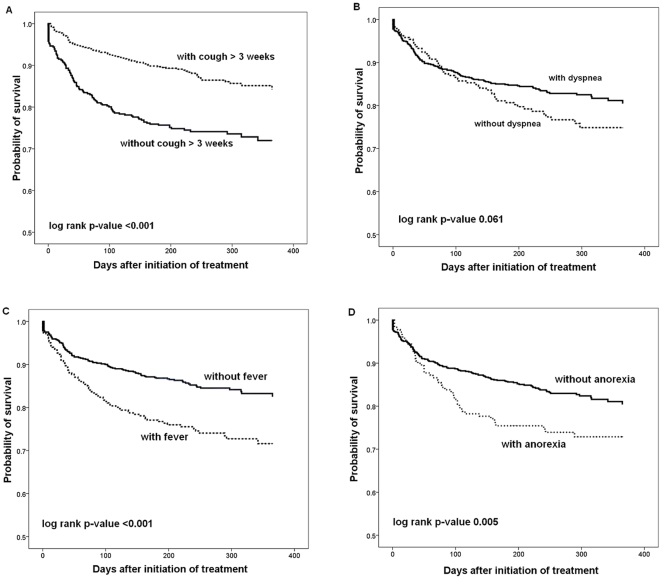
Kaplan-Meier survival curves of pulmonary tuberculosis patients stratified by the presence or absence of clinical symptoms/signs. (A) Chronic cough > three weeks. (B) Dyspnea. (C) Fever. (D) Anorexia. Significances were tested using the log-rank test.

Kaplan-Meier survival curves according to the presence or absence of clinical symptoms/signs are shown in Figure 3. Patients who presented with fever, anorexia, and absence of chronic cough of > 3 weeks had higher mortalities (p<0.001 for fever, p = 0.005 for anorexia, and p<0.001 for chronic cough). An absolute difference in survival between patients with and without chronic cough was evident after the initiation of anti-TB treatment.

### The characteristics of patients with early mortality

The demographic characteristics of patients with early (within 30 days) and late (after 30 days) mortality are shown in Table 3. As compared with late mortality, patients with early mortality are less likely to be sputum smear positive (RR: 0.63, 95% CI: 0.41–0.97), to present with chronic cough > 3 weeks (RR: 0.51, 95% CI: 0.32–0.82) and dyspnea (RR: 0.50, 95% CI: 0.28–0.88). Other clinical factors, including age, gender, underlying comorbidities, drug susceptibility profiles, and radiological presentations were comparable between patients with early or late mortality. Multivariate Poisson regression analysis identified that the initial presentations of chronic cough > 3 weeks and dyspnea were independently associated with a lower probability of early mortality within 30 days.

**Table 3:** Poisson regression analysis for early mortality (within 30 days) prediction by demographic characteristics and clinical presentations.a,b

	All mortality patients, n = 195	Mortality status		Unadjusted RR (95% CI) c		Adjusted RR (95% CI) d	
		Early mortality, n = 62	Late mortality, n = 133	RR (95% CI)	P value	RR (95% CI)	P value
Mean Age (SD)	76.2 (12.6)	77.2 (15.3)	75.7 (11.2)	1.01 (0.99–1.03)	0.53		
Initial sputum smear positive	91 (46.7%)	22 (35.5%)	69 (51.9%)	0.63 (0.41–0.97)	0.033		
Previous TB history	18 (9.2%)	3 (4.8%)	15 (11.3%)	0.50 (0.17–1.43)	0.16		
Malignancy	61 (31.3%)	17 (27.4%)	44 (33.1%)	0.83 (0.52–1.33)	0.43		
Renal insufficiency	23 (11.8%)	11 (17.7%)	12 (9%)	1.61 (0.99–2.62)	0.08		
Post gastrectomy	14 (7.2%)	4 (6.5%)	10 (7.5%)	0.89 (0.38–2.10)	0.79		
Isoniazid resistance	23 (11.8%)	4 (6.5%)	19 (14.3%)	0.52 (0.21–1.29)	0.11		
Rifampicin resistance	11 (5.6%)	1 (1.6%)	10 (7.5%)	0.27 (0.04–1.80)	0.10		
MDR	10 (5.1%)	1 (1.6%)	9 (6.8%)	0.30 (0.05–1.97)	0.13		
Beijing strain infection	112 (57.4%)	35 (56.5%)	77 (57.9%)	0.96 (0.63–1.45)	0.85		
Cavity formation	22 (11.3%)	7 (11.3%)	15 (11.3%)	1.00 (0.52–1.92)	1.00		
Lobar/segmental consolidation	170 (87.2%)	54 (87.1%)	116 (87.2%)	0.99 (0.54–1.83)	0.98		
Chronic cough	83 (42.6%)	17 (27.4%)	66 (49.6%)	0.51 (0.32–0.82)	0.003	0.56 (0.33–0.98)	0.041
Hemoptysis	35 (17.9%)	16 (25.8%)	19 (14.3%)	1.59 (1.03–2.46)	0.05		
Dyspnea	59 (30.3%)	11 (17.7%)	48 (36.1%)	0.50 (0.28–0.88)	0.009	0.51 (0.27–0.98)	0.043
Fever	83 (42.6%)	27 (43.5%)	56 (42.1%)	1.04 (0.69–1.57)	0.85		
Malaise	41 (21.0%)	16 (25.8%)	25 (18.8%)	1.31 (0.83–2.05)	0.26		
Anorexia	50 (25.6%)	11 (17.7%)	39 (29.3%)	0.63 (0.35–1.10)	0.09		

a The data are presented as n (%) unless otherwise stated.

b Only mortality cases are included for analysis.

c Unadjusted risk ratios and 95% confidence intervals for clinically relevant variables.

d Results of stepwise selection for a fitted Poisson Regression model (Goodness-of-fit, Chi square = 9.55, p = 0.008). Variables were included in the final model for p<0.05 and were excluded if p<0.1.

TB, tuberculosis; MDR, multi-drug resistance; RR, risk ratio; CI, confidence interval.

## Discussion

Despite effective first line anti-TB drugs, pulmonary tuberculosis remains a disease with a high mortality rate. The mortality rates for pulmonary tuberculosis reported in previous studies ranged from 5% to 30%, depending upon underlying co-morbidities, disease severity, and HIV-infection status of the included patients [2], [6], [13], [14]. In the present study, 19.7% of the patients died before completing the treatment, which was relatively higher as compared with previous reports in non-HIV endemic areas. The higher mortality rate among the patients we enrolled was probably due to older age and more underlying co-morbidities, as the study was performed in referral medical centers. In line with previous reports [3], [15], a higher proportion of TB patients died in the early phase of commencing treatment. Nearly one-third of the deaths occurred within 30 days, and more than half of the deaths occurred within 60 days after initiating anti-TB treatment. Our observations highlight the importance of identifying predictors that are associated with mortality in the start of anti-TB treatment.

The risk factors associated with mortality have been extensively evaluated before. Age, co-morbidities of malignancy, renal disease, and respiratory disorders, HIV infection, and malnutrition are all well-established clinical predictors of mortality in tuberculosis patients [4]–[6], [8], [14], [16]. For HIV-infected patients, CD4 T cell counts and HIV viral loads have been identified as significant factors of mortality in tuberculosis [2], [17]. In addition, low socioeconomic status, multidrug resistance, and delayed diagnosis have also been reported to be significant predictors of mortality [18]–[21]. In the present study, we found that older age and the underlying co-morbidities of malignancy and renal insufficiency were independent demographic characteristics associated with mortality, whereas drug susceptibility profiles and genotypes of MTB isolates were not. Drug resistance, especially multidrug resistance tuberculosis (MDRTB), is widely reported to be associated with mortality [3], [9], [21]. However, the associations between drug resistance and mortality were insignificant in our report. In Taiwan, MDRTB patients are encouraged to be transferred to referral TB centers and routinely requested to join a Direct Observation Therapy/Short Course (DOTS) program. The treatment regimen is adjusted according to the complete second line anti-TB drugs susceptibility profiles and is under the stringent supervision of CDC. All of these measures help to improve the treatment outcome of MDRTB patients. Our results indicated that the major determinants for mortality among PTB patients in Taiwan, a TB endemic area with high medical accessibility and abundant medical resources, were those factors related to the underlying conditions of the patients, and not bacterial factors.

The mortality rates were comparable between PTB patients with and without HIV-infections in the present study. Taiwan is not a HIV endemic area and HIV testing is not routinely performed in general tuberculosis patients. The estimated HIV prevalence rate of adults in Taiwan was 0.16% in 2008. By comparison, the estimated HIV prevalence rate was 0.64% in overall newly diagnosed TB patients and 1.8% in newly diagnosed TB patients with available HIV testing results [22]. The HIV prevalence rate is likely to be underestimated as the HIV test is not routinely done for our patients. The insignificant correlation between HIV infection and mortality deserves further verification in future studies.

All of the patients we enrolled were culture-proved cases and, therefore, the genotyping data were available for these patients. The Beijing genotype is distributed worldwide and is the predominant MTB strain in Eastern Europe and Southeast Asia [23], [24]. Although previous studies reported higher treatment failures and relapse rates for patients infected with the Beijing strain [25], [26], the mortality rates were comparable between patients infected with Beijing and non-Beijing strains in the present study. Based on these results, the impact of the Beijing strain on mortality is probably limited in Taiwan. However, the clinical characteristics, such as drug resistance, associated with the Beijing strain vary between geographic areas and its role in treatment outcomes may also be different [23]. Further studies are required to clarify the impact of the Beijing strain on mortality.

The association between clinical presentations and mortality remains an issue of controversy. Kourbatova et al. reported bilateral lung involvement, cavitary lesions on radiograms, and symptoms lasting > 4 weeks as independent risk factors of mortality for tuberculosis patients [9]. Hoa et al. also found that tuberculosis patients with prolonged coughs had an increased risk of death [10]. However, Venkatarama et al. reported that only symptomatic dyspnea was independently associated with mortality for hospitalized tuberculosis patients [8]. Low at al. identified the absence of cough and cavitary lesions on radiograms as significant predictors of mortality by multivariate analysis [3].

In our analysis, the presence of fever, anorexia, and the absence of chronic cough of > 3 weeks were clinical presentations that were independently associated with mortality. The associations of radiological features for predicting mortality were limited. More importantly, we found that the absence of respiratory symptoms, including chronic cough and dyspnea, was the sole independent predictor for early mortality within 30 days. The differences in patient settings, demographic characteristics, and HIV-infection status may have contributed to the inconsistent findings between our results and those of previous reports.

Our findings also provide important information for physicians in clinical practice. The presence of constitutional symptoms, such as fever and anorexia, may represent higher disease severity and associated with increased risk of death. Meanwhile, respiratory symptoms/signs are important clues that can remind clinical physicians of the possibility of pulmonary diseases. The absence of typical respiratory symptoms may lessen clinical suspicion and result in delayed diagnosis of pulmonary tuberculosis. A delayed diagnosis can lead to increased risks of mortality [27]. However, information regarding the durations of presenting symptoms/signs was not collected in the study design. Therefore, the association between delayed diagnosis and mortality cannot be readily analyzed, and this made it difficult for us to clarify the interaction between presenting symptoms/signs and mortality. However, delays in diagnosis remain the most probable cause for a worse outcome in patients without respiratory symptoms.

Possible predictors of early mortality as compared with late mortality among PTB patients have rarely been analyzed before. Interestingly, we found that only the absence of respiratory symptoms, but neither age nor co-morbidities, was an independent predictor of mortality within 30 days. The findings again emphasize the importance of early diagnosis and early treatment in the improving outcome of PTB patients. The proportions of previous TB history were comparable between patient with early and late mortality in the present study. However, the impact of TB reaction remains an issue that deserves discussion as critical patients probably are more likely to have TB reactivation and this leads to early mortality. This study was carried out in a TB endemic area and unrecognized previous TB infection is highly possible with uncertain incidence. The proportion of previous TB infection is obviously underestimated. However, a history of previous TB infection does not necessarily indicate the presence of reactivation, as reinfection could be a major cause of recurrent TB after previous cure [28]. Without genotyping profiles of previous infection, it is difficult to differentiate TB reactivation from TB reinfection in these patients. Further studies are needed to elucidate this issue.

Several independent risk factors for death were identified in the present study. Unfortunately, all of these were un-modifiable factors. What actions can we take when managing these high risk patients? Irrespective of the cause of mortality, treating the underlying comorbidities of patients as best as possible is definitely important. It would be helpful to encourage the patients to join a DOTS program to enhance compliance of anti-TB treatment. However, the crucial step probably lies in early anti-TB treatment; and early treatment mostly depends on early diagnosis. Maintaining a high degree of clinical suspicion in order to diagnosis PTB early is of pivotal importance to improve the treatment outcome of PTB patient.

Our study had several limitations. Five of the six participating hospitals were regional referral medical centers. More patients with more co-morbidities and higher disease severities may have been included in the present study and, as a result, the overall mortality rate may have been overestimated. However, this also shows that our findings can be reliably applied to elderly PTB patients with multiple co-morbidities. Information regarding the durations of presenting symptoms prior to PTB diagnosis was not collected in the study design. This limitation made it more difficult to interpret the association between symptoms/signs and mortality. We did not divide deaths as being due to either TB or other non-TB causes in the study design because it is not always easy to identify the impact of tuberculosis on mortality. All-cause mortality is more objective and more applicable to clinical practice. As the study area not an HIV endemic area, HIV testing was not routinely conducted in the present study. This also limits the ability of our findings to be applied in non-HIV-endemic areas.

In conclusion, our study found that nearly one-third of the mortality cases among PTB patients occurred within 30 days of initiating anti-TB treatment. Irrespective of the cause of mortality, older age and the underlying co-morbidities of malignancy and renal insufficiency were demographic characteristics that associated with death. Regarding clinical presentations, the presence of fever, anorexia, and the absence of chronic cough were independent predictors of death. As compared with late mortality, the absence of respiratory symptoms, including chronic cough and dyspnea, were significant factors associated with early mortality within 30 days. In contrast, the associations of radiological features with early and overall mortalities were limited.
